# Supplementary material for: Neonatal Subcutaneous BCG Vaccination Decreases Atherosclerotic Plaque Number and Plaque Macrophage Content in ApoE−/− Mice
Source: Biology (Basel). 2022 Oct 15;11(10):1511. doi: 10.3390/biology11101511 (PMC9599032; doi:10.3390/biology11101511)
Supplement: Supplementary file 1 [file biology-11-01511-s001.zip › KeyResourcesTable_Bekkering.pdf]

Major Resources Table

In order to allow validation and replication of experiments, all essential research materials listed in the Methods should be included in the Major Resources Table below. Authors are encouraged to use public repositories for protocols, data, code, and other materials and provide persistent identifiers and/or links to repositories when available. Authors may add or delete rows as needed.

Animals (in vivo studies)

Species	Vendor or Source	Background Strain	Sex	Persistent ID / URL
ApoE-/- Mouse	Jackson	C57Bl/6/J	Both Male and Female	

Antibodies

Target antigen	Vendor or Source	Catalog #	Working concentration	Persistent ID / URL
Rat monoclonal (CI:A3-1)anti Mouse F4/80	Abcam	Ab6640	0,05 mg/ml	https://www.abcam.com/f480-antibody-cia3-1-macrophage-marker-ab6640.html
Goat anti Rat IgG, mouse adsorbed	Vector Laboratories	BA-9401	1 ug/ml	https://vectorlabs.com/products/antibodies/biotinylated-goat-anti-rat-igg-mouse-adsorbed

Other

Description	Source / Repository	Persistent ID / URL
Western fast food diet	Specialty feeds Australia; SF00-219	https://www.specialtyfeeds.com/diets/sf00-219/
Middlebrook 7H9 Broth Base	Sigma-Aldrich; M0178	https://www.sigmaaldrich.com/NL/en/product/sial/m0178
Middlebrook ADC growth supplement (10%)	Sigma-Aldrich; M0553	https://www.sigmaaldrich.com/NL/en/product/sial/m0553
Cholesterol E test	Fujifilm Wako Pure Chemical Corporation; 439-17501	
Triglyceride E test	Fujifilm Wako Pure Chemical corporation; 432-40201	
Dextraslip 50	Sigma-Aldrich; D8787-1G	https://www.sigmaaldrich.com/NL/en/product/sial/d8787
Mayer's Hematoxylin Solution	Amber Scientific; 24821-500	https://www.polysciences.com/german/mayers-hematoxylin-40779
Eosin 1% Aqueous Solution	Amber Scientific	
Oil Red O working solution	Sigma Aldrich #MAK194	
Fluorescence Mounting Medium	Dako #S3022380-2	
Fiji software	Image J version 2.0	
3% H2O2 peroxidase blocking solution	Dako S2023	
Serum-free protein block	Dako, X0909	
Wash buffer immunohistochemistry	Dako K8024	

DOI [to be added]

Streptavidin HRP	Dako P0397	
DAB chromogen in substrate buffer	Dako K5007	
Haematoxylin	Dako, S3301	
Leica CV mount	Leica, 14046430011	

DOI [to be added]
