# Supplementary material for: Neonatal Subcutaneous BCG Vaccination Decreases Atherosclerotic Plaque Number and Plaque Macrophage Content in ApoE−/− Mice
Source: Biology (Basel). 2022 Oct 15;11(10):1511. doi: 10.3390/biology11101511 (PMC9599032; doi:10.3390/biology11101511)
Supplement: Supplementary file 1 [file biology-11-01511-s001.zip › Supplementary Table S1.pdf]

Supplementary Table S1

**Cause of death**	**Group**
Pups eaten by mother (n=2)	Saline
Malocclusions (n=1)	Saline
Distended abdomen due to bowel obstruction (n=1)	Saline
Hydrocephalus (n=1)	BCG
Found dead in cage (n=1)	Saline

Suppl table S1. Summary of exclusion of mice before 16 weeks.
