# Supplementary material for: Neonatal Subcutaneous BCG Vaccination Decreases Atherosclerotic Plaque Number and Plaque Macrophage Content in ApoE−/− Mice
Source: Biology (Basel). 2022 Oct 15;11(10):1511. doi: 10.3390/biology11101511 (PMC9599032; doi:10.3390/biology11101511)
Supplement: Supplementary file 1 [file biology-11-01511-s001.zip › Supplementary Table S2.pdf]

Supplementary Table S2. Overview of atherosclerosis model systems, diets, BCG (dose and form), delivery routes and effects on atherosclerosis. Chol, cholesterol; CFU, Colony Forming Units; BCG, Bacille Calmette-Guérin; s.c, subcutaneous; i.v., intravenous; i.n., intranasal; i.p., intraperitoneal; LDLR, Low-Density Lipoprotein Receptor; ApoE, Apolipoprotein E

Model system	Diet	Form of BCG	Dose	Delivery Route	Effect on atherosclerosis (and time of measurement)	Ref
Mouse LDLR-/- and ApoE-/-	Chow with 15% lard and 0.5% chol resp. Chow	Extended freeze-drying	100 ug	4-6 s.c. injections in base of tail	**30 weeks atherosclerosis**<br>Reduced size of lesions<br>Reduced serum cytokines	9
Mouse ApoE3Leiden.CETP	Western diet 0.1% chol	Live BCG, human dose	1 human dose $5x10^6$ CFU	Single i.v. injection	**6 weeks atherosclerosis**<br>Delayed lesion formation in aortic root<br>Reduces plasma non-HDL-c<br>Reduced foam cell formation	10
Mouse LDLR-/-	Western diet (21%fat, 0.2%chol)	Live BCG	$0.3\text{-}3x10^6$ CFU	I.n.	**16 week atherosclerosis**<br>Increased lesions (by Oil Red)<br>Increased CD4/CD8 T cell ratio	11
Yarkon Chicken	Ad lib commercial feed (2% chol and 1% chol)	Live BCG (Glaxo)	0.5 human dose	S.c. or i.p.<br>3 different timings: (1) 8 weeks before diet, (2) Together with diet, (3) 5 weeks after diet start	**Chicken age 42**<br>(1) IP No effect<br>(2) IP slight increase atherosclerosis<br>(3) IP decreased atherosclerosis<br>1. SC decreased atherosclerosis<br>2. SC No change<br>3. SC No change	12
New Zealand White rabbits	1% cholesterol diet with adapt to maintain plasma chol.	Live BCG	1 + 0.5 human dose (4-$13x10^6$ CFU)	2x s.c.	**Injection at 12 weeks**<br>Aortic IMT increase<br>Activated leukocytes	13
Mouse ApoE-/-		Heat killed BCG	0.01-1 mg	I.p.	**2 weeks after BCG, 16-week old mice**<br>1mg BCG increased calcified lesions<br>0.01mg no effect	15
