# Supplementary figures and images for: Neonatal Subcutaneous BCG Vaccination Decreases Atherosclerotic Plaque Number and Plaque Macrophage Content in ApoE−/− Mice
Source: Biology (Basel). 2022 Oct 15;11(10):1511. doi: 10.3390/biology11101511 (PMC9599032; doi:10.3390/biology11101511)

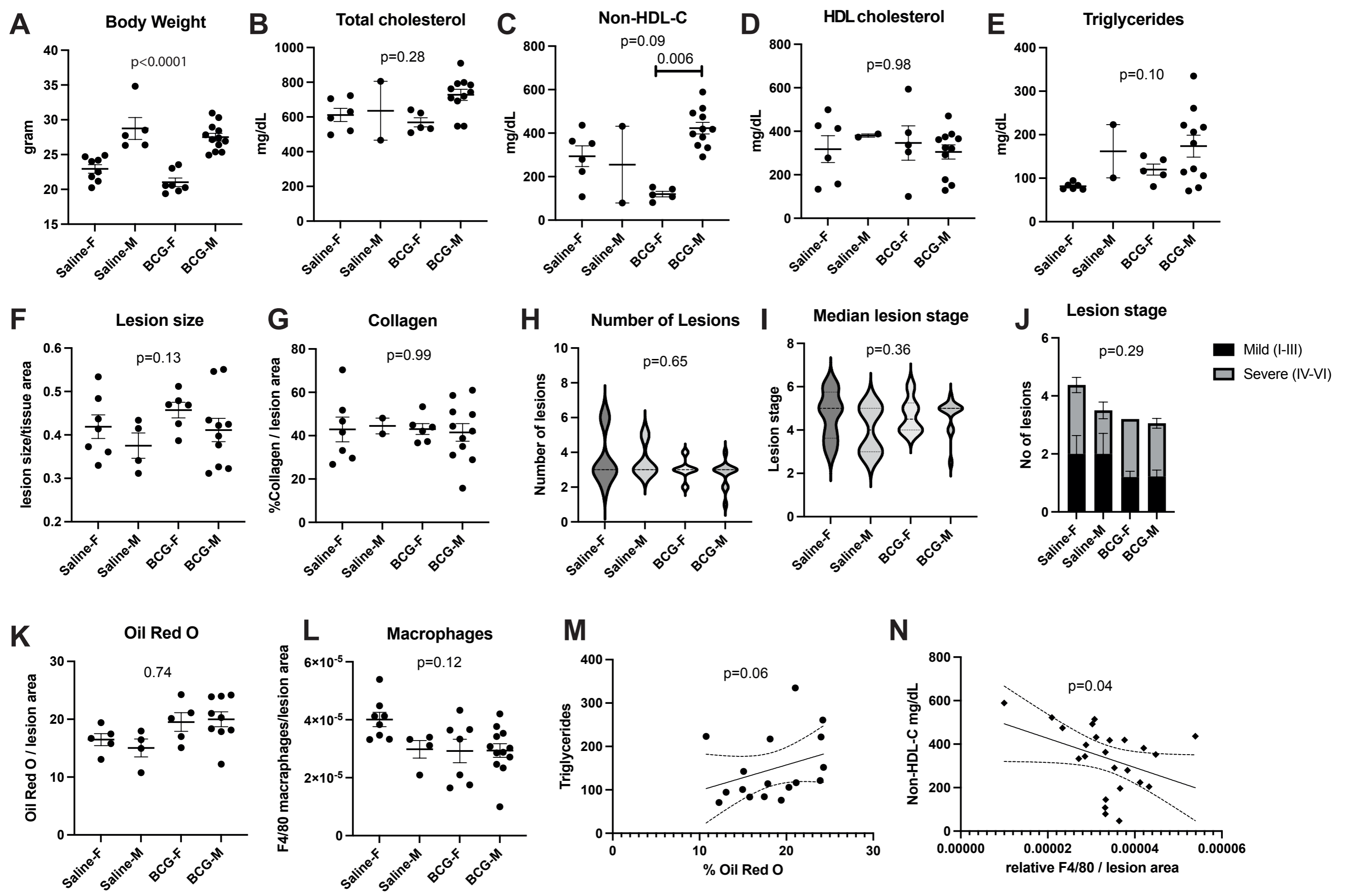

Supplement: Supplementary file 1 [file biology-11-01511-s001.zip › Supplementary Figure S1_sex differences.pdf]
